# A Syntenic Cross Species Aneuploidy Genetic Screen Links RCAN1 Expression to β-Cell Mitochondrial Dysfunction in Type 2 Diabetes

**DOI:** 10.1371/journal.pgen.1006033

**Published:** 2016-05-19

**Authors:** Heshan Peiris, Michael D. Duffield, Joao Fadista, Claire F. Jessup, Vinder Kashmir, Amanda J. Genders, Sean L. McGee, Alyce M. Martin, Madiha Saiedi, Nicholas Morton, Roderick Carter, Michael A. Cousin, Alexandros C. Kokotos, Nikolay Oskolkov, Petr Volkov, Tertius A. Hough, Elizabeth M. C. Fisher, Victor L. J. Tybulewicz, Jorge Busciglio, Pinar E. Coskun, Ann Becker, Pavel V. Belichenko, William C. Mobley, Michael T. Ryan, Jeng Yie Chan, D. Ross Laybutt, P. Toby Coates, Sijun Yang, Charlotte Ling, Leif Groop, Melanie A. Pritchard, Damien J. Keating

**Affiliations:** 1 Department of Human Physiology and Centre for Neuroscience, Flinders University, Adelaide, South Australia, Australia; 2 Lund University Diabetes Centre, Malmö, Sweden; 3 Islet Biology Laboratory, Department of Anatomy and Histology and Centre for Neuroscience, Flinders University, Adelaide, South Australia, Australia; 4 Discipline of Medicine, University of Adelaide, Adelaide, South Australia, Australia; 5 Metabolic Remodelling Laboratory, Metabolic Research Unit, School of Medicine, Deakin University, Geelong, Australia; 6 Metabolism and Inflammation Program, Baker IDI Heart and Diabetes Institute, Melbourne, Australia; 7 Queen's Medical Research Institute, University of Edinburgh, Edinburgh, United Kingdom; 8 Centre for Integrative Physiology, University of Edinburgh, Edinburgh, United Kingdom; 9 Mary Lyon Centre Pathology, MRC Harwell, Harwell Oxford Science Park, Oxford, United Kingdom; 10 Department of Neurodegenerative Disease, UCL Institute of Neurology, London, United Kingdom; 11 Francis Crick Institute, Mill Hill, London, United Kingdom; 12 Department of Medicine, Imperial College London, London, United Kingdom; 13 Department of Neurobiology and Behaviour, University of California, Irvine, Irvine, California, United States of America; 14 Department of Neurosciences School of Medicine, University of California, San Diego, San Diego, California, United States of America; 15 Department of Biochemistry and Molecular Biology, Monash University, Melbourne, Victoria, Australia; 16 Diabetes and Metabolism Division, Garvan Institute of Medical Research, Darlinghurst, Sydney, New South Wales, Australia; 17 Clinical and Experimental Transplantation Group, Royal Adelaide Hospital, North Terrace, Adelaide, South Australia, Australia; 18 Animal Experiment Center, Animal Biosafety Level-III Laboratory, Wuhan University, Wuhan, China; 19 South Australian Health and Medical Research Institute, Adelaide, Australia; Centre for Cancer Biology, SA Pathology, AUSTRALIA

## Abstract

Type 2 diabetes (T2D) is a complex metabolic disease associated with obesity, insulin resistance and hypoinsulinemia due to pancreatic β-cell dysfunction. Reduced mitochondrial function is thought to be central to β-cell dysfunction. Mitochondrial dysfunction and reduced insulin secretion are also observed in β-cells of humans with the most common human genetic disorder, Down syndrome (DS, Trisomy 21). To identify regions of chromosome 21 that may be associated with perturbed glucose homeostasis we profiled the glycaemic status of different DS mouse models. The Ts65Dn and Dp16 DS mouse lines were hyperglycemic, while Tc1 and Ts1Rhr mice were not, providing us with a region of chromosome 21 containing genes that cause hyperglycemia. We then examined whether any of these genes were upregulated in a set of ~5,000 gene expression changes we had identified in a large gene expression analysis of human T2D β-cells. This approach produced a single gene, *RCAN1*, as a candidate gene linking hyperglycemia and functional changes in T2D β-cells. Further investigations demonstrated that *RCAN1* methylation is reduced in human T2D islets at multiple sites, correlating with increased expression. RCAN1 protein expression was also increased in db/db mouse islets and in human and mouse islets exposed to high glucose. Mice overexpressing RCAN1 had reduced *in vivo* glucose-stimulated insulin secretion and their β-cells displayed mitochondrial dysfunction including hyperpolarised membrane potential, reduced oxidative phosphorylation and low ATP production. This lack of β-cell ATP had functional consequences by negatively affecting both glucose-stimulated membrane depolarisation and ATP-dependent insulin granule exocytosis. Thus, from amongst the myriad of gene expression changes occurring in T2D β-cells where we had little knowledge of which changes cause β-cell dysfunction, we applied a trisomy 21 screening approach which linked RCAN1 to β-cell mitochondrial dysfunction in T2D.

## Author Summary

Mitochondrial dysfunction and reduced insulin secretion are key features of β-cell dysfunction in Type 2 diabetes (T2D). Down syndrome (DS) is a genetic disorder caused by trisomy of chromosome 21 that also displays β-cell mitochondrial dysfunction and reduced insulin secretion in humans. Given these similarities in β-cell dysfunction in T2D and DS, we developed a trisomy 21 screening method to identify genes that may be important in T2D. This approach used different DS mouse models combined with human gene expression data from T2D β-cells. From this we identified a single candidate, Regulator of calcineurin 1 (RCAN1). High RCAN1 expression occurs in human and mouse T2D islets. Increased RCAN1 expression in mice reduced β-cell mitochondrial function and ATP availability, and this has negative implications for multiple ATP-dependent steps in glucose-stimulated insulin secretion.

## Introduction

Type 2 diabetes (T2D) is a complex metabolic disorder characterised by elevated blood glucose levels. Pancreatic β-cell dysfunction and reduced insulin output in the presence of insulin resistance is the primary cause of T2D. The mechanisms leading to a switch from β-cell compensation during the early stages of insulin resistance to β-cell failure in the latter stages remain unknown. Studies from human T2D islets provide the most direct evidence regarding the nature of such β-cell changes. Reduced β-cell mass and insulin content is observed in T2D [1], but these are not insurmountable given the capacity of sulphonylureas, GLP-1 agonists or bariatric surgery to restore insulin secretion and plasma glucose in T2D patients. Clearly alternative pathways exist to drive β-cell dysfunction and reduced glucose-stimulated insulin secretion (GSIS). For example, oxidative stress is increased in human T2D β-cells and negatively correlates with GSIS impairment [2]. T2D β-cells also display marked mitochondrial dysfunction; characterised by a reduced respiratory response to glucose [3] in association with lower ATP levels [4]. Given that mitochondrial function is central to oxidative stress, ATP production and GSIS in β-cells, and that these are major defects in T2D β-cells, identifying the genes responsible for β-cell mitochondrial dysfunction is essential to further our understanding of the mechanisms controlling β-cell function.

As one approach to identifying causative genes, several genome-wide association studies (GWAS) have compared gene expression changes in healthy and T2D human patients (see [5] for full details) and gene array and proteomic studies have been conducted on T2D islets [6,7]. The largest such study involved 89 donors and identified 4,920 gene expression changes using RNA Sequencing in T2D islets [8]. However, identifying which of these changes are functionally relevant to β-cell dysfunction in T2D is a significant challenge.

Interestingly, islets derived from fetal Down syndrome (DS) tissue exhibit β-cell mitochondrial dysfunction, low ATP levels and reduced insulin secretion [9]. We have therefore exploited the phenotypes shared by β-cells derived from DS and T2D islets in an attempt to detect functionally relevant genes in human islets that underlie β-cell dysfunction in T2D. Using this approach we identified a single lead candidate, a gene called Regulator of calcineurin 1 (RCAN1), which is overexpressed in T2D islets and when overexpressed in mouse islets, causes β-cell mitochondrial dysfunction and reduced ATP production to inhibit insulin secretion.

## Results

### A DS screening approach identifies that RCAN1 is overexpressed in human and mouse Type 2 diabetes islets

**Fig 1:**
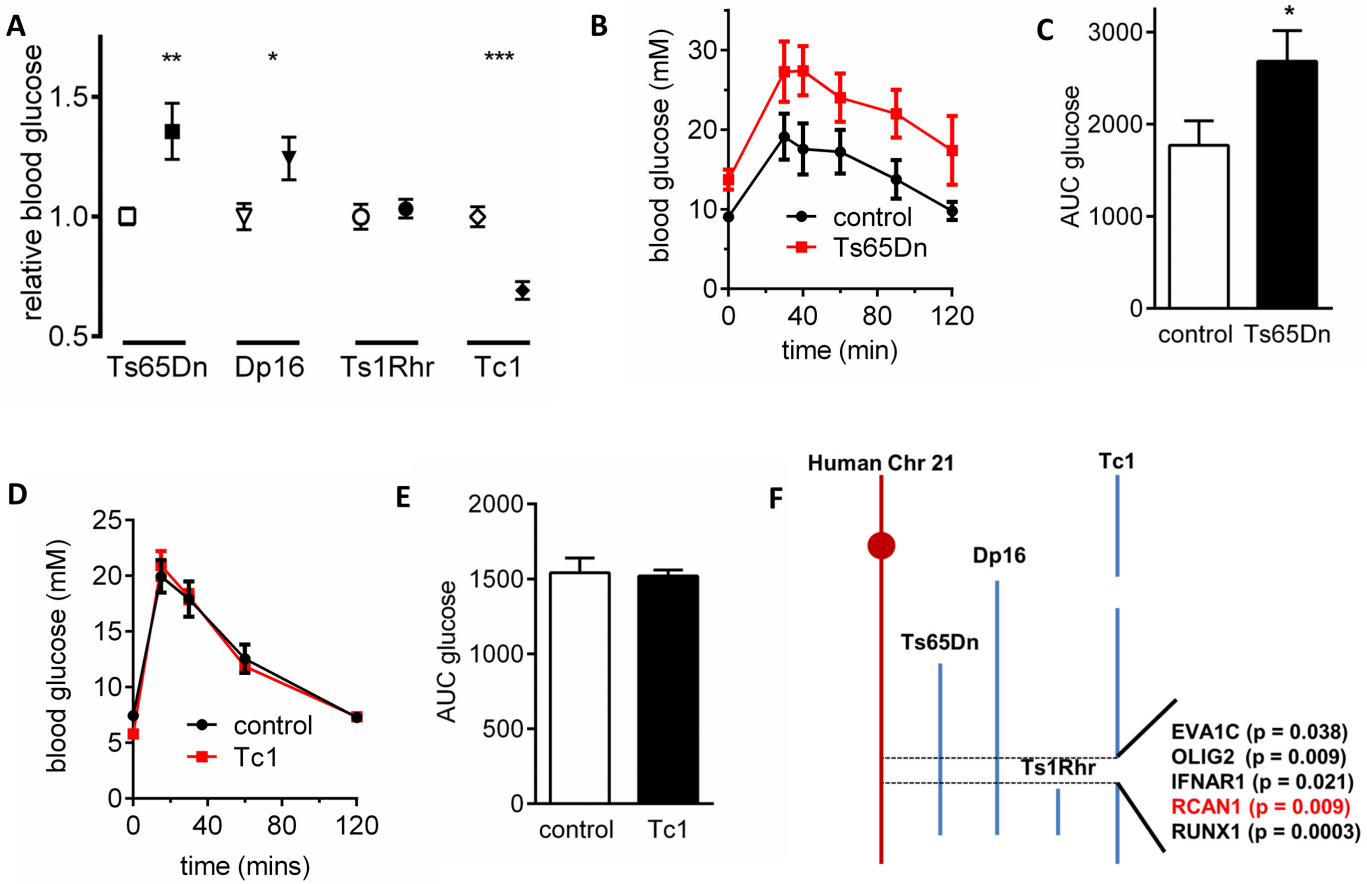
A DS screening approach identifies a region of chromosome 21 associated with hyperglycemia. (A) Fasting blood glucose in Ts65Dn (n = 14), Dp16 (n = 11), Ts1Rhr (n = 12) and Tc1 (n = 23) mice (filled symbols) and their respective controls (open symbols). (B) Glucose tolerance test in Ts65Dn (n = 5) and control (n = 6) mice and (C) area under the curve analysis. (D) Glucose tolerance test in Tc1 (n = 9) and control (n = 8) mice and (E) area under the curve analysis. (F) Illustration of the trisomic regions in these DS models used and the 5 genes associated with hyperglycemia in DS that are up-regulated in human T2D islets. *p < 0.05, **p < 0.01.

To screen for chromosome 21 genes that may contribute to diabetes, measured fasting blood glucose levels (in mM) were measured in 4 mouse models of DS; Ts65Dn, Dp16, Ts1Rhr and Tc1 (Fig 1A). These transgenic mouse lines either have a partial or whole trisomy of mouse chromosome 16 (largely homologous to human chromosome 21) or contain an incomplete freely segregating human chromosome 21 (Tc1) (Fig 1F). We found that Ts65Dn (control 8.0 ± 0.3, n = 17 vs Ts65Dn 10.9 ± 0.9, n = 14, *p* < 0.01) and Dp16 (control 9.7 ± 0.5, n = 10 vs Dp16 12.1 ± 0.9, n = 11, *p* < 0.05) mice were hyperglycaemic, Ts1Rhr mice were normoglycaemic (control 11.1 ± 0.6, n = 18 vs Ts1Rhr 11.5 ± 0.4, n = 12), and Tc1 mice were hypoglycaemic (control 13.6 ± 0.6, n = 23 vs Tc1 9.4 ± 0.5, n = 23, *p* < 0.001). Ts65Dn mice (Fig 1B and 1C) demonstrated poorer glucose tolerance (measured by intraperitoneal glucose tolerance tests (IPGTT)) but no change was observed in the Tc1 mouse line (Fig 1D and 1E). These data indicated that a subset of chromosome 21 genes contributed to the development of glucose intolerance and hyperglycaemia. Mapping the trisomic regions of chromosome 21 unique to Ts65Dn and Dp16 mice amongst these lines identified a region of 38 candidate genes that contains genes that must contribute to the hyperglycaemic phenotype (Fig 1F).

We next used RNA Seq data from human islets from a Swedish study obtained from 89 donors (77 non-diabetic (ND, HbA1c <6.5%) and 12 T2D (HbA1c >6.5%) [8] to identify whether any of these 38 genes have increased expression in T2D islets. Five genes were significantly up regulated; *EVA1C* (p = 0.038), *OLIG2* (p = 0.009), *IFNAR1* (p = 0.021), *RCAN1* (p = 0.009) and *RUNX1* (p = 0.0003) (Fig 1F). EVA1C is a Slit receptor involved in Robo-mediated axonal guidance [10], OLIG2 regulates spinal cord oligodendrocyte and motor neuron development [11], IFNAR1 is an interferon receptor with no role in the initiation or progression of diabetes [12] and RUNX1 is involved in haematopoiesis [13]. Only RCAN1 has any known role in affecting mitochondrial function [14] or insulin secretion when either chronically overexpressed in mouse islets [15] or transiently transfected into a mouse β-cell line [16].

**Fig 2:**
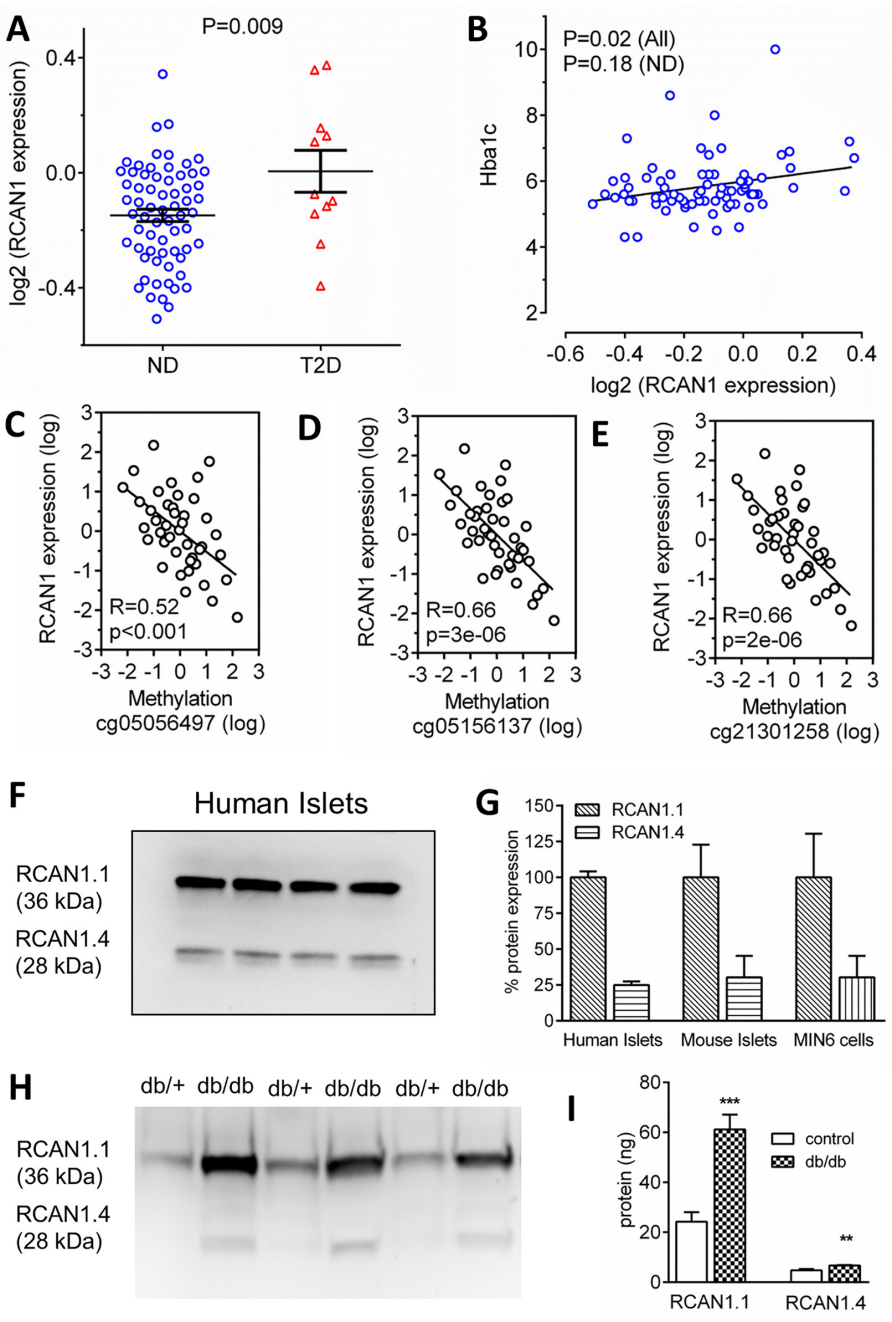
RCAN1 expression and methylation in human and mouse Type 2 diabetic islets. (A) RCAN1 gene expression in human non-diabetic (ND, n = 77, blue symbols) and type 2 diabetic islets (T2D, n = 12, red symbols). (B) RCAN1 expression vs donor HbA1c (n = 89). RCAN1 expression and methylation status at sites (C) cg05056497, (D) cg05156137 and (E) cg21301258 are strongly correlated. (F) RCAN1.1 and RCAN1.4 protein expression in human islets. (G) Quantification of RCAN1 islet protein expression in human islets (n = 3), mouse islets (n = 5) and mouse MIN6 β-cells (n = 3). (H) RCAN1.1 and RCAN1.4 protein expression in control (db/+) and db/db islets. (I) Quantification of (G) (n = 6 control, n = 5 db/db). **p < 0.01, ***p < 0.001.

*RCAN1* is highly abundant in human islets, being in the top 13% most highly expressed genes across the genome [8]. In human T2D islets, gene expression of *RCAN1* was 153% of that in ND islets (Fig 2A). *RCAN1* expression correlated significantly with the clinical measure of long-term glycaemic status, glycosylated haemoglobin (HbA1c, Fig 2B) and BMI (S1A Fig) across all samples. When *RCAN1* expression was separated across HbA1c categories, we found that expression in islets from HbA1c >6.5% (T2D) was higher than that in healthy islets (HbA1c <6%) but not different to those with impaired glucose tolerance (HbA1c 6–6.5%) (S1B Fig), indicating that islet RCAN1 expression increases after insulin resistance has occurred. Furthermore, this human islet data was consolidated by global islet genome expression data from an earlier study [17] that profiled gene expression in islets from an obesity-induced diabetes-resistant mouse strain and from a diabetes-susceptible mouse strain before (4 weeks of age) and after (10 weeks of age) the onset of diabetes. This mouse islet data demonstrated that mouse islet *RCAN1* expression is correlated with increased plasma triglycerides (S1C Fig) and increased body weight (S1D Fig), both of which are associated with T2D in humans.

To understand more about *RCAN1* in human T2D islets, we analysed previously published data using the Infinium 450k array to examine global changes in DNA methylation in human T2D islets [18]. Compared to the non-diabetic group, *RCAN1* had the fifth largest difference in methylation across the entire analysed genome in T2D islets, and the largest difference in methylation of all analysed chromosome 21 genes [18]. Additionally, of all 16 analysed *RCAN1* methylation sites on the 450k array, 3 show a significant negative correlation across all samples between methylation status and *RCAN1* expression (Fig 2C–2E). Importantly, these 3 sites also displayed significant reductions in methylation in T2D islets from 51.1 ± 1.8% in non-diabetic islets (n = 34) to 40.2 ± 3.1% in T2D islets (n = 15, p<0.01, data is mean ± SEM) at site cg05156137, 57.9 ± 1.9% vs 48 ± 3.2% (p<0.01) at cg21301258 and 42.3 ± 1.5% vs 29.5 ± 1.9% (p<0.001) at cg05056497 [18]. Thus, our data demonstrated that RCAN1 expression is linked to hyperglycaemia in DS mice, it correlated in islets with a worsening metabolic profile in obese mice, and it is increased in human T2D islets. Furthermore, RCAN1 methylation at three different sites correlated with RCAN1 expression in human islets and methylation status at these sites is reduced in T2D islets. This combination of multiple data sets strongly supports the concept that RCAN1 expression is increased in T2D β-cells.

**Fig 3:**
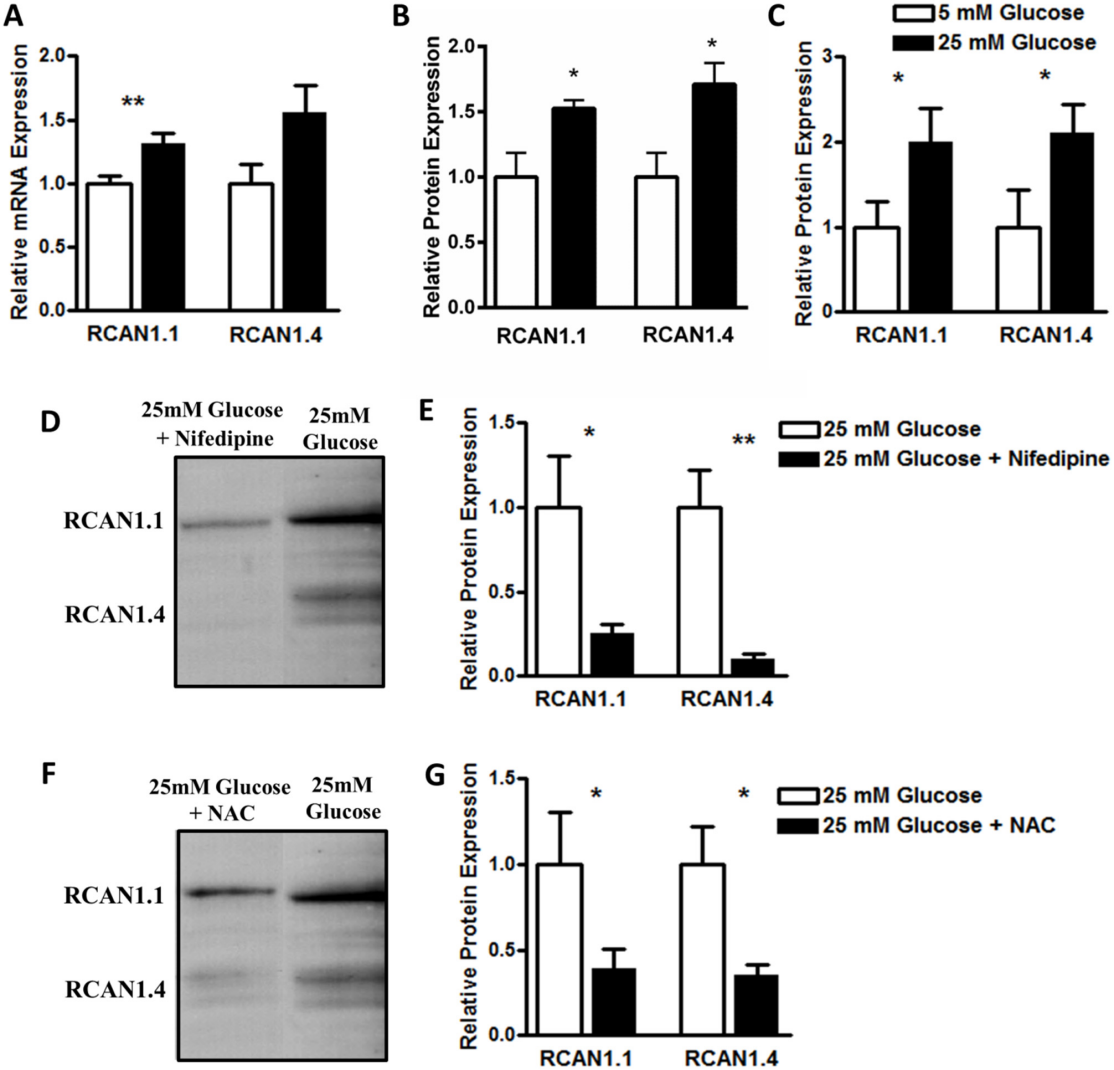
β-cell RCAN1 expression is increased under hyperglycemic conditions via Ca^2+^ and oxidative stress. **(A)** RCAN1.1 expression (mRNA) is significantly higher in human pancreatic islets cultured at 25 mM glucose (black bars) when compared to islets cultured at 5.5 mM glucose (white bars) (n = 5 experiments). **(B)** RCAN1.1 and RCAN1.4 expression (protein) is significantly higher in mouse pancreatic islets cultured at 16.7 mM glucose (black bars) when compared to islets cultured at 5.5 mM glucose (white bars). (n = 4 experiments). **(C)** RCAN1.1 and RCAN1.4 expression (protein) is significantly higher in MIN6 cells cultured at 25 mM glucose for six days (black bars) compared to cells cultured at 5 mM glucose (white bars) for the same time period, (n = 5 experiments). **(D)** Representative Western blot showing reduced RCAN1.1 and RCAN1.4 expression in MIN6 cells cultured at 25 mM glucose +Nifedipine for 6 days compared to cells cultured at 25 mM glucose for the same time period. **(E)** Quantification of Western blot images, (n = 5 experiments). **(F)** Representative Western blot showing reduced RCAN1.1 and RCAN1.4 expression in MIN6 cells cultured at 25 mM glucose +NAC for 6 days compared to cells cultured at 25 mM glucose for the same time period. **(G)** Quantification of Western blot images, (n = 5 experiments). Data represents the mean ± SEM, *p<0.05, **p<0.01.

We next confirmed whether human islets express the RCAN1 protein. RCAN1 has two isoforms called RCAN1.1 and RCAN1.4 (S2D Fig). Each differs in their start exon but both contain common exons 5, 6 and 7. Exon 7 contains the calcineurin binding motif [19] and both isoforms are thought to have shared functions as inhibitors of calcineurin. Western blot analysis revealed that RCAN1.1 and RCAN1.4 isoforms were both present (Fig 2F), with the RCAN1.1 isoform expression ~4 fold higher, similar to mouse islets and the mouse MIN6 β-cell line, an insulinoma cell line derived from a transgenic mouse expressing the large T-antigen of SV40 in pancreatic β-cells (Fig 2G). We observed a significant increase in RCAN1.1 and RCAN1.4 protein (Fig 2H) in islets from the leptin receptor deficient T2D mouse model, db/db (12 week old males, db/+ weight = 22.5 ± 1.1 g, plasma glucose = 8.5 ± 0.3 mM, db/db weight = 46.6 ± 1.1 g, plasma glucose = 24.1 ± 1.4 mM). RCAN1.1 expression was ~10 times that of RCAN1.4 in db/db islets (Fig 2I). Prolonged exposure to high glucose also induced RCAN1 expression in human islets (Fig 3A), mouse islets (Fig 3B) and MIN6 cells (Fig 3C). This induction was reversed when Ca^2+^ entry was reduced with the L-type Ca^2+^ channel blocker nifedipine (Fig 3D and 3E) or by inhibiting oxidative stress with the antioxidant N-acetylcysteine (NAC) (Fig 3F and 3G). Thus, increased Ca^2+^ and oxidative stress both induced β-cell RCAN1 expression under hyperglycaemic conditions and RCAN1.1 is the major RCAN1 isoform in human and mouse β-cells.

### Overexpression of RCAN1 in mice reduces *in vivo* GSIS

**Fig 4:**
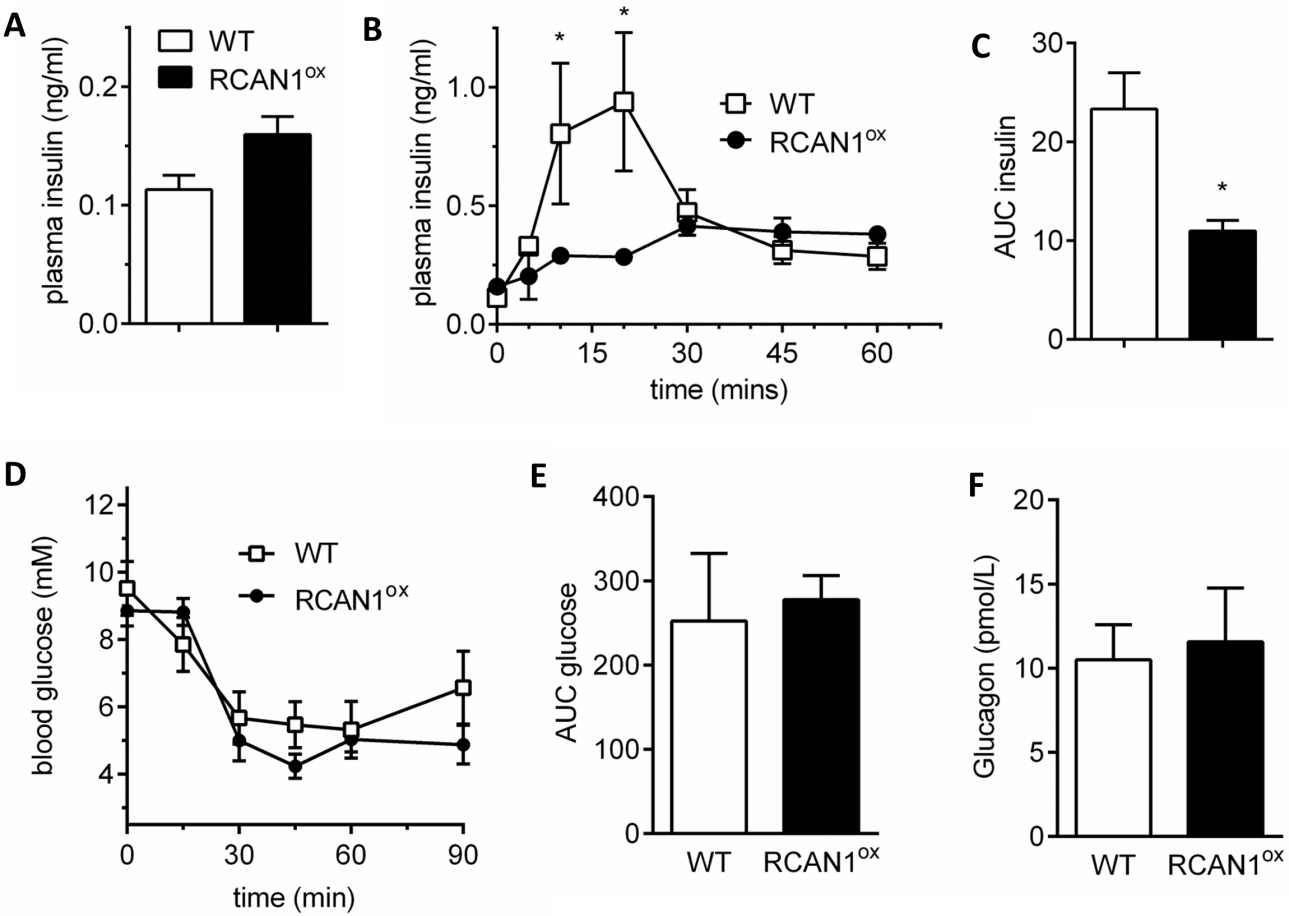
GSIS is reduced *in vivo* in RCAN1^ox^ β-cells. (A) Plasma insulin is similar in WT and RCAN1^ox^ mice (n = 3). (B) *in vivo* GSIS is lower in RCAN1^ox^ mice as defined by (C) reduced area under the curve (AUC) for insulin secretion over 1 hour (n = 3). (D) Insulin sensitivity in WT (n = 4) and RCAN1^ox^ (n = 5) mice is not different as defined by (E) AUC analysis. (F) Plasma glucagon levels are similar in WT (n = 5) and RCAN1^ox^ (n = 4) mice. Data represents the mean ± SEM, *p<0.05.

We measured *in vivo* GSIS in mice overexpressing RCAN1.1 (henceforth referred to as RCAN1^ox^) as reduced GSIS is a hallmark in T2D individuals [14,15,20], and as RCAN1.1 is the major β-cell RCAN1 isoform with the greatest increase in db/db islets. Basal plasma insulin levels were not different (Fig 4A) but *in vivo* GSIS is reduced in RCAN1^ox^ mice (Fig 4B and 4C). This is similar to findings demonstrating that transient overexpression of RCAN1 in a β-cell line reduced *in vitro* GSIS [16]. This reduced *in vivo* GSIS in RCAN1^ox^ mice is not due to increased insulin sensitivity, as these mice demonstrated no change in insulin tolerance (Fig 4D and 4E) or in plasma glucagon levels (Fig 4F).

### Overexpression of RCAN1 causes β-cell mitochondrial dysfunction

**Fig 5:**
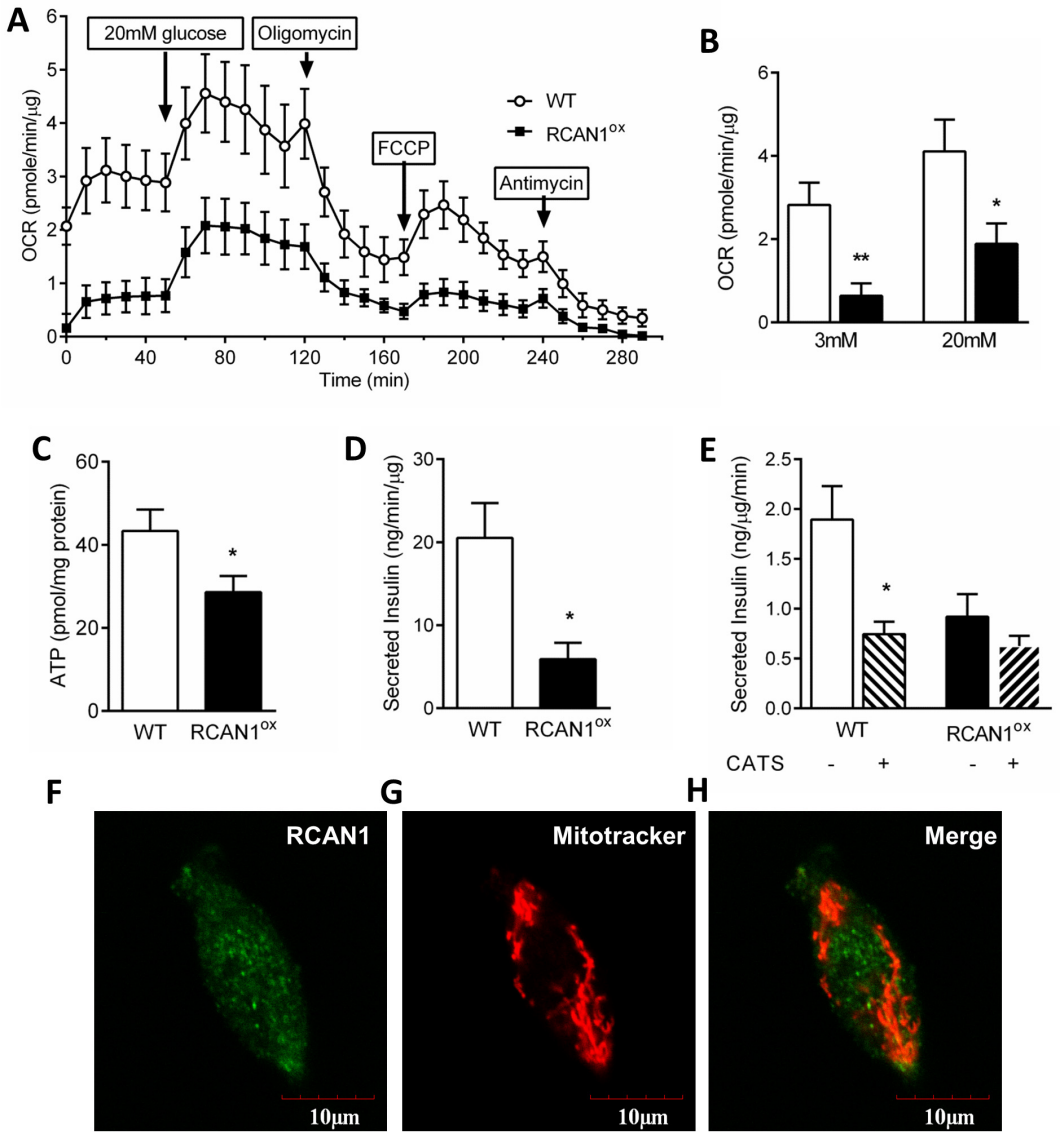
Mitochondrial respiratory output is reduced in RCAN1^ox^ islets. (A) Oxygen consumption rate (OCR) in WT (n = 5) and RCAN1^ox^ (n = 6) islets under various conditions. (B) OCR in 3 mM or 20 mM glucose. (C) Islet ATP levels (n = 7 WT and n = 4 RCAN1^ox^). (D) Methyl-succinate-induced (10 mM) insulin secretion (n = 4/group). (E) Effect of carboxyatractyloside (CATS, 200 μm) on insulin secretion in response to 20 mM glucose (n = 4–7). Immunocytochemical labelling of (F) RCAN1, (G) mitochondria (Mitotracker Red) and (H) both in MIN6 cells. *p < 0.05, **p < 0.01.

We then tested whether increasing RCAN1 affects β-cell mitochondrial function, as the focus of this study is to identify potential regulators of mitochondrial dysfunction in T2D β-cells. Islets from RCAN1^ox^ mice have double the RCAN1.1 gene expression and a 2.5 fold increase in protein level [14], similar to the increase in RCAN1.1 protein expression that we report here in db/db islets. Islet respiration (oxygen consumption rate) was assessed in RCAN1^ox^ and wild-type (WT) islets (Fig 5A). At both 3mM and 20mM glucose RCAN1^ox^ islets had a significantly lower basal oxygen consumption rate (3mM WT 2.82 ± 0.54 vs RCAN1^ox^ 0.64 ± 0.30 pmoles/min/μg protein and 20mM WT 4.11 ± 0.76 vs RCAN1^ox^ 1.89 ± 0.49 pmoles /min/μg protein, Fig 5B). Uncoupled respiration due to proton (H^+^) leak (measured in the presence of oligomycin) was also significantly lower in RCAN1^ox^ islets (WT 1.28 ± 0.34 vs RCAN1^ox^ 0.48 ± 0.26 pmoles/min/μg protein, S3A Fig). Basal mitochondrial respiration was significantly decreased and respiration due to ATP turnover showed a trend towards reduction in RCAN1^ox^ islets (S3B and S3C Fig) demonstrating a consistent respiratory defect with RCAN1 overexpression across multiple aspects of mitochondrial function.

Unsurprisingly, ATP levels were reduced in RCAN1^ox^ islets (Fig 5C). To understand whether these changes are due to alterations upstream of the mitochondrial electron transport chain or could be directly attributed to mitochondrial dysfunction, we stimulated complex II of the electron transport chain directly with methyl succinate. This resulted in significant insulin secretion from WT islets but far less in RCAN1^ox^ islets (Fig 5D), indicating a respiratory defect within the mitochondria downstream of complex I. Since RCAN1 interacts with the mitochondrial ATP translocator, ANT, in *Drosophila* neurons [21], we used the ANT inhibitor carboxyatractyloside (CAT) to test whether the effect of RCAN1 on mitochondrial function in β-cells is mediated through changes in ANT function. CAT significantly reduced GSIS in WT islets, but had no effect in RCAN1^ox^ islets (Fig 5E). RCAN1 does not have a classical N-terminal mitochondrial localisation signal [22] and immunocytochemical co-localisation studies further demonstrate that RCAN1 does not localise to mitochondria in MIN6 cells (Fig 5F–5H). While some overlap was observed due to the nature of co-localisation studies, we believe the data does not support a mitochondrial localisation of RCAN1. No fluorescence was detected when the primary antibody was replaced by either saline or non-immune rabbit serum, and used with the secondary antibody as negative controls.

### RCAN1 regulates mitochondrial membrane potential

**Fig 6:**
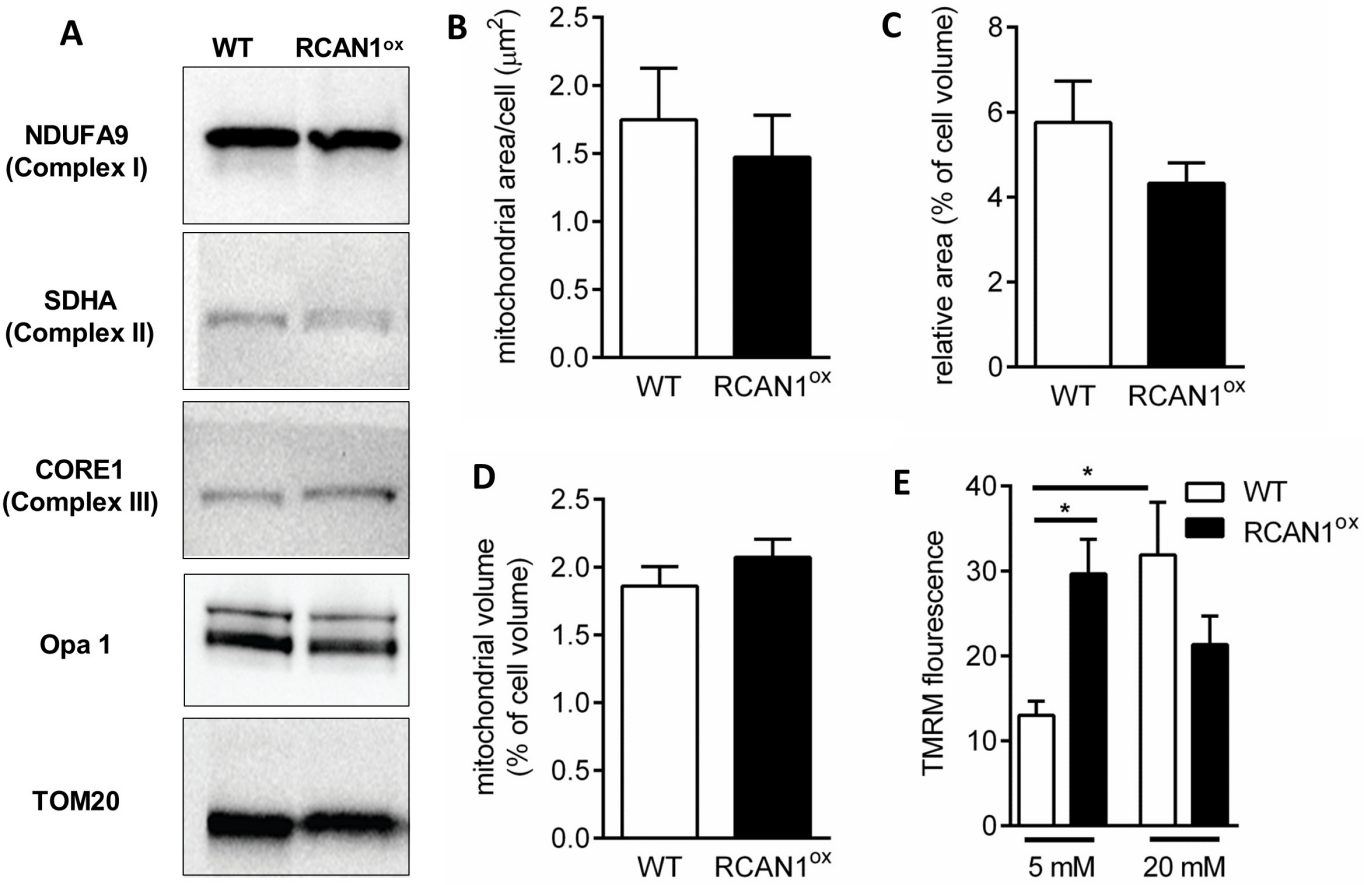
RCAN1 effects mitochondrial membrane potential, not volume. (A) Representative western blots of wild type (WT) and RCAN1^ox^ islet protein lysates probed against NDUFA9, SDHA, CORE1, Opa1 and TOM20. Electron microscopy analysis of (B) total mitochondrial area and (C) relative area in WT (n = 8 β-cells) and RCAN1^ox^ (n = 6 β-cells). (D) TMRM staining in live cells further demonstrates a lack of difference in mitochondrial volume between WT (n = 43 cells) and RCAN1^ox^ (n = 46 cells). (E) Mitochondrial membrane potential measured as mean cell TMRM fluorescence in low and high glucose in WT and RCAN1^ox^ cells (n = 17–21 cell/group). Data represents the mean ±SEM, *p<0.05.

To further understand how mitochondrial output is diminished in RCAN1^ox^ β-cells, we first measured the protein expression of various mitochondrial markers. No changes in the expression of TOM20 (marker of total mitochondrial protein), NDUFA9 (complex I), SDHA (complex II), or CORE1 (complex III) were observed, and a lack of OPA1 cleavage infers mitochondrial fission was unaltered (Fig 6A). Electron microscopy analysis of mitochondrial area in β-cells further demonstrated no change in the absolute (Fig 6B) or relative (Fig 6C) area of mitochondria in RCAN1^ox^ β-cells. We observed the same outcome when measuring mitochondrial volume using the mitochondrial marker tetramethylrhodamine methyl ester (TMRM) in live cells (Fig 6D). We observe increased TMRM fluorescence in RCAN1^ox^ β-cells at rest, indicative of mitochondrial hyperpolarisation (Fig 6E). Furthermore, while a normal mitochondrial hyperpolarisation in response to glucose [23] was observed in WT cells, no such response occurred in RCAN1^ox^ cells (Fig 6E). Thus, the mitochondrial electrochemical gradient is altered in RCAN1^ox^ β-cells and does not change in response to high glucose.

### Increased RCAN1 expression reduces glucose-induced membrane depolarization in β-cells

**Fig 7:**
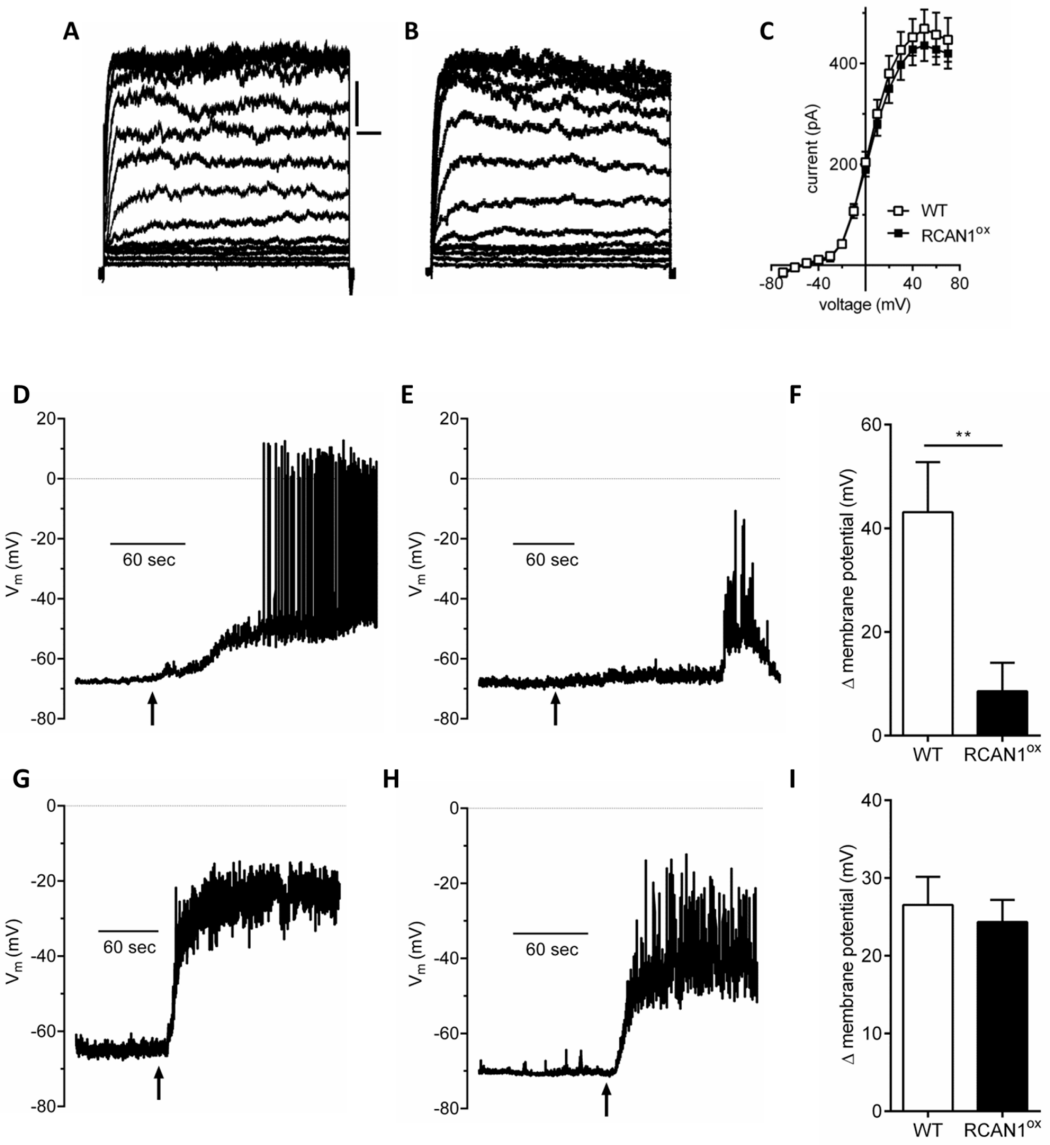
Glucose-dependent membrane depolarisation is reduced in RCAN1^ox^ β-cells. Voltage clamp recordings in (A) WT and (B) RCAN1^ox^ β-cells. Scale bar in A is 20ms and 20pA in A and B. (C) Current-voltage relationship in WT (n = 59) and RCAN1^ox^ (n = 39) β-cells. Current clamp recordings from (D) WT and (E) RCAN1^ox^ β-cells in response to 20mM glucose (arrow). (F) Membrane potential change in response to 20mM glucose in WT (n = 6) and RCAN1^ox^ (n = 7) β-cells (**p < 0.01). Current clamp recordings from (G) WT and (H) RCAN1^ox^ β-cells in response to tolbutamide (100μM, arrow). (I) Membrane potential change in response to tolbutamide in WT (n = 6) and RCAN1^ox^ (n = 6) β-cells.

Having established that mitochondrial function and ATP output are compromised in RCAN1^ox^ β-cells, we next tested whether this is functionally relevant for specific ATP-dependent components of the GSIS pathway. Mitochondria are central to ATP production in response to high glucose in β-cells. This triggers closure of plasma membrane K_ATP_ channels, membrane depolarisation, Ca^2+^ entry and insulin secretion. Perforated patch whole cell voltage clamp recordings from single β-cells were used to measure K^+^ currents in WT (Fig 7A) and RCAN1^ox^ (Fig 7B) β-cells. This established that the average current-voltage relationships were identical in these cells (Fig 7C). In the presence of 20mM glucose, the K^+^ current amplitude produced by this same voltage pulse protocol was reduced in WT β-cells and caused a rightward shift in the current-voltage relationship near the reversal potential, indicative of membrane depolarisation (S4A Fig). However such a change was diminished in RCAN1^ox^ β-cells (S4B Fig). To measure membrane potential directly, current clamp recordings, still obtained in the perforated patch clamp configuration to maintain the endogenous intracellular ATP concentration, were undertaken. These demonstrated that WT (Fig 7D) and RCAN1^ox^ (Fig 7E) β-cells were both depolarised and fire action potentials in response to high glucose and had similar resting membrane potentials (WT -67.4 ± 1.4 mV, n = 7, RCAN1^ox^ -65.3 ± 3.4 mV, n = 10). However, the amount of glucose-induced membrane depolarisation was less in RCAN1^ox^ β-cells (*p* < 0.01, Fig 7F). Addition of the K_ATP_ channel antagonist, tolbutamide, caused membrane depolarisation in WT (Fig 7G) and RCAN1^ox^ β-cells (Fig 7H) to the same extent (Fig 7I). Similarly in voltage clamp mode, tolbutamide caused equal changes in K^+^ current amplitude and the estimated reversal potential in both WT (S4C Fig) and RCAN1^ox^ (S4D Fig) β-cells. Thus, the decreased ATP production in RCAN1^ox^ β-cells reduced glucose-induced closure of plasma membrane K_ATP_ channels to limit glucose-induced membrane depolarization.

### Reduced ATP availability in RCAN1^ox^ β-cells decreases depolarization-induced insulin exocytosis

**Fig 8:**
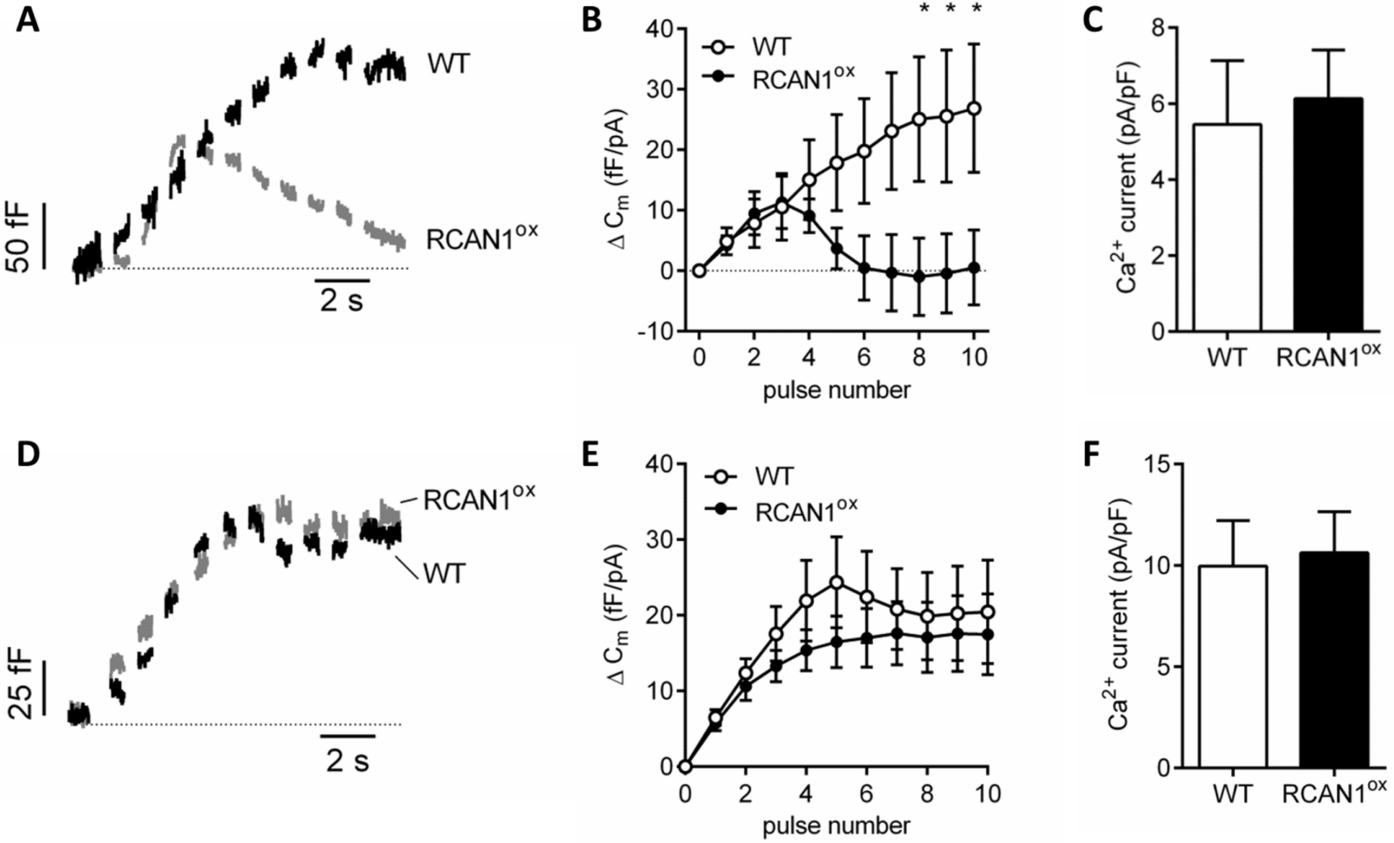
Depolarization-induced insulin secretion is reduced in RCAN1^ox^ β-cells due to low ATP availability. (A) Perforated patch membrane capacitance trace from WT and RCAN1^ox^ β-cells. (B) Average capacitance change at each depolarising pulse in WT (n = 14) and RCAN1^ox^ (n = 18) β-cells, *p < 0.05. (C) Ca^2+^ current size from these recordings. (D) Membrane capacitance trace from WT and RCAN1^ox^ β-cells in whole cell mode with 3mM ATP added to the pipette solution. (E) Average capacitance change at each depolarising pulse in WT (n = 13) and RCAN1^ox^ (n = 19) β-cells. (F) Ca^2+^ current size from these recordings.

As ATP is also required for vesicle transport in β-cells [24], we utilized membrane capacitance measurements to test if RCAN1 overexpression negatively affects insulin exocytosis. We initially used the perforated patch clamp mode to maintain endogenous intracellular ATP concentrations. A series of ten depolarizing pulses resulted in an increase in membrane capacitance in WT (Fig 8A) and RCAN1^ox^ β-cells due to insulin exocytosis but this secretion was not sustained in RCAN1^ox^ β-cells (Fig 8B). This was not due to altered Ca^2+^ current size (Fig 8C). Given ATP production is low in RCAN1^ox^ β-cells; we then punctured the cell membrane using the whole cell patch clamp approach in order to introduce an equal amount of ATP (3mM) into the β-cell cytosol in both groups via the pipette solution. Under these conditions we observed robust secretion in WT and RCAN1^ox^ β-cells (Fig 8D) that is similar in both groups (Fig 8E). Ca^2+^ current size is the same in these groups (Fig 8F). Thus, a lack of ATP in RCAN1^ox^ β-cells has negative effects directly on vesicle fusion.

## Discussion

In this study we attempted to identify genes that regulate mitochondrial function in β-cells and underlie mitochondrial dysfunction and reduced GSIS in T2D β-cells. This approach produced a single candidate, RCAN1, which is overexpressed in human T2D islets. We validated our screening approach by establishing that higher RCAN1 levels caused mitochondrial dysfunction resulting in low ATP levels in β-cells. This reduced ATP availability has direct functional consequences on glucose-stimulated membrane depolarisation as well as depolarisation-induced insulin exocytosis. Further investigation revealed that increased RCAN1 hyperpolarises the mitochondrial membrane and blunts the respiratory output of β-cell mitochondria. Thus, this approach enabled the identification of a single candidate gene, from 4,920 genes with altered expression in T2D islets [8], that is capable of causing mitochondrial dysfunction and reduced insulin secretion.

T2D is a complex multi-systemic metabolic disorder with β-cell dysfunction at its core. Islets isolated from T2D patients have lower ATP levels [4] and elevated ROS accumulation that correlates with the impairment of GSIS [25] which is reversed in human T2D islets pre-treated with anti-oxidants [26]. Such data highlights the central role of oxidative stress and impaired mitochondrial function in T2D β-cell dysfunction.

Islets from DS individuals display fragmented mitochondria and reduced insulin secretion [9] and similar changes are seen in human T2D β-cells. While the mechanisms underlying these mitochondrial changes in DS β-cells remain poorly defined, DS individuals also have mitochondrial dysfunction and increased oxidative stress in a number of other cell types [9,27,28]. The early onset of mitochondrial dysfunction in fetal DS β-cells is indicative of genetic mechanisms driving this phenotype. Our observation that the trisomy 21 mouse models, Ts65Dn and Ts16, are hyperglycaemic, and that Ts65Dn mice have impaired glucose tolerance, indicates that some mouse chromosome 16 genes regulate blood glucose levels and potentially β-cell function. This hyperglycemia may well be due to reduced β-cell output, similar to that observed in human DS islets [9]. As Tc1 and Ts1Rhr mice are not hyperglycaemic, and Tc1 mice have normal glucose tolerance, we were able to refine the list of candidate genes to 38. Of these, five were overexpressed in T2D islets; *EVA1C*, *OLIG2*, *IFNAR1*, *RCAN1 and RUNX1*. *RCAN1* was of particular interest when focusing on mitochondrial function and insulin secretion. We note that we have not confirmed that RCAN1 expression is increased in human DS islets or in islets from mouse models of trisomy 21. However given that neuronal expression of RCAN1 is almost double that of normal [29], we assume that this is similar in mouse models triplicating RCAN1 such as Ts65Dn and Tc1. Given that the altered mitochondrial morphology observed in human DS β-cells [9], is not seen in our β-cells overexpressing RCAN1, chromosome 21 genes other than RCAN1 may also cause some of the pathological changes in DS β-cells. We must emphasize here that the DS screening approach was not used to provide a guide on DS genes that cause hyperglycemia or β-cell dysfunction, but rather as a tool to help identify genes important in β-cell dysfunction in T2D from a study that had yielded almost 5000 gene expression changes.

The relationship between DS and diabetes is both poorly studied and highly complex. While the incidence of T1D is increased in the DS population [30,31], few publications exist regarding the incidence of T2D. One such study indicates an increased incidence of T2D in DS individuals [32] and high fasting blood glucose and insulin resistance are more prevalent in the DS population [33,34]. However the increased prevalence of obesity in this population needs to be considered. The diagnosis of young DS individuals with T1D is not always based on an increased incidence of β-cell autoantibodies [31], and clarification in a larger study will be needed to clearly identify whether these cases are truly autoimmune T1D or a genetically-caused early onset form of diabetes associated with chromosome 21 genes being overexpressed. It is also worth noting that fine mapping of a region on chromosome 21 that shows linkage to T1D contains RCAN1 [35]. Given that increased hypoinsulinemia has been long reported in the DS population [36] and that β-cell dysfunction and low insulin release is seen even in fetal DS islets [9], DS individuals may have the capacity to cope with this through other genetically induced changes that increase, for example, peripheral insulin sensitivity. The hypoglycaemia observed in the Tc1 mouse model indicates there may be some chromosome 21 genes that would provide beneficial effects on glycemic control in diabetes. This idea is reinforced in the human insulin-dependent DS population who require a lower dose of insulin compared with age-matched insulin-dependent diabetics [37]. Further study into whether some chromosome 21 genes could be responsible for improved insulin sensitivity or reduced insulin secretion are worthy of future investigation.

RCAN1 is a stress-induced protein known to regulate mitochondrial function in neurons [14,21,38]. RCAN1 overexpression increases mitochondrial ROS in neurons and pancreatic islets, and reduces GSIS [14,15]. As such, it was our lead candidate from the results of our screening approach. The DNA methylation data in human T2D islets adds key mechanistic insight to explain how *RCAN1* expression is increased in T2D β-cells. Reduced *RCAN1* methylation was found to correlate with increased *RCAN1* expression at three different sites, and it was only at these sites that the methylation status was reduced in T2D islets. What drives these methylation changes in β-cell RCAN1 during T2D is an important question worth addressing in the future. Our Western blot data from db/db mouse islets illustrates that RCAN1 protein expression increases in T2D islets, and that RCAN1.1 is 10 times more highly expressed than RCAN1.4 in db/db islets. Such data is a good illustration that gene changes may not reflect protein expression given the 50% increase in *RCAN1* gene expression in T2D islets vs. 3 fold increased RCAN1.1 protein expression in db/db islets. It also highlights the fact that genome-wide expression screens may miss important targets that have small gene, but large protein, expression changes. As such, approaches such as GWAS, as well as our DS-based approach, will be limited in their scope in this respect. RCAN1.1 and RCAN1.4 expression were also elevated in response to glucose in MIN6 cells. This was driven by Ca^2+^ and oxidative stress, consistent with the mechanism of RCAN1 induction in neurons [39]. Thus, we demonstrate that RCAN1 expression is linked to hyperglycaemia in DS mice, that it correlates in islets with a worsening metabolic profile in obese mice, that it is increased in human T2D islets, that RCAN1 methylation at three methylation sites correlates with RCAN1 expression in human islets and that methylation status at these sites is reduced in T2D islets. This is further consolidated by protein expression data demonstrating not only that RCAN1.1 and RCAN1.4 increases in db/db islets but that chronic high glucose increases expression of both RCAN1 isoforms in islets through a mechanism that involves increased Ca^2+^ and oxidative stress. Ca^2+^ entry is thought to be increased in T2D β-cells due to chronic high glucose levels and oxidative stress is linked to the degree of GSIS impairment in human T2D islets. This data strongly supports the concept that RCAN1 expression increases in T2D β-cells and that this is driven my cell stresses that are relevant to the pathogenesis of β-cell failure in T2D.

The effects on β-cell function that we attribute to RCAN1 overexpression in our transgenic mice are consolidated by the findings of others using independent models. This includes the finding that transient overexpression of RCAN1 reduces GSIS in a β-cell line [16] and significantly, that β-cell number and islet size are reduced, as reported in our RCAN1^ox^ line [15], in mice overexpressing RCAN1 created independently of our study [40]. Thus is appears unlikely that off-target effects related to transgene insertion in our RCAN1^ox^ mice drive the phenotypes we observe here.

We demonstrate that increased RCAN1 inhibits ATP production and mitochondrial function. The lack of succinate-induced insulin secretion clearly identifies defective mitochondrial respiration in RCAN1^ox^ β-cells, but exactly what drives this is unclear. ANT exports ATP out of the mitochondrial matrix while importing ADP. Disruption of ANT reduces cellular ATP levels and inhibits the electron transport chain, resulting in reduced oxygen consumption rates, such as we observe in RCAN1^ox^ islets. In one respect, our data is consistent with findings in *Drosophila* neurons that RCAN1 interacts with and blocks ANT activity when overexpressed and reduces cytosolic ATP levels [21]. Our finding that mitochondria are hyperpolarized in RCAN1^ox^ β-cells could be explained by reduced ANT output because a decrease in cellular ATP levels, resulting in increased substrate entry into the electron transport chain. However, if ATP is trapped in the mitochondria due to defective nucleotide transport, ATP synthase, and therefore respiration, will be inhibited. As electrons continue to enter the electron transport chain, protons will continue to be pumped into the inner membrane space and membrane potential will increase as we observed in RCAN1^ox^ β-cells in 3 mM glucose. This situation also occurs in our WT β-cells in the presence of increased glucose, except that as ATP is exported from the mitochondria in these cells, respiration increases. However, as we do not observe a clear mitochondrial localisation of RCAN1 in β-cells a direct interaction between RCAN1 and ANT seems unlikely. We suggest alternatively that inhibiting ANT activity in RCAN1^ox^ β-cells did not reduce insulin secretion because the mitochondria is already hyperpolarised and added glucose cannot drive further respiration in RCAN1^ox^ β-cells.

We observe RCAN1 MIN6 cell localisation in both the cytoplasm and nucleus. This is somewhat in contrast to similar experiments showing little β-cell nuclear localisation [15]. RCAN1 is normally expressed in both the nucleus and cytoplasm, with the degree of nuclear localisation being cell-type dependent [41]. RCAN1 nuclear localisation is significantly reduced in the presence of activated calcineurin [42]. As calcineurin activity is regulated acutely by calcium, differences in RCAN1 localisation could readily be caused by small differences in experimental conditions such as culture media, temperature, buffer capacity of solutions and any stress placed upon the cells. This should be kept in mind when comparing cytosolic and nuclear RCAN1 localisation. No evidence suggests that these factors affect mitochondrial localisation of RCAN1, but it is worth considering given that this has been reported in *Drosophila* neurons [21].

The mitochondrial respiratory defects we observe in RCAN1^ox^ β-cells are not associated with altered mitochondrial morphology. The expression of TOM20, a marker of total mitochondrial protein, is unchanged and electron microscopy analysis and mitochondrial staining indicate that mitochondrial mass is unchanged in β-cells overexpressing RCAN1. The reduced oxygen consumption rate we observe at baseline in RCAN1^ox^ islets is therefore not due to reduced mitochondrial mass. Some reduction in mitochondrial size is reported in RCAN1^ox^ β-cells but this is far less than that reported in neurons [14,21,38]. Whether such differences are strain or cell type dependent is unknown but our data shows that overt morphological changes are not required for RCAN1 to cause mitochondrial dysfunction. Mitochondrial and cytoplasmic calcineurin activity increases in response to changes in cytosolic Ca^2+^ in β-cells [43]. While the functional roles of altered calcineurin activity in different intracellular locations is not clear, increased RCAN1 would be expected to suppress at least some of them, potentially including mitochondrial oxidative phosphorylation. As RCAN1 is not present in β-cell mitochondria, other possibilities such as changes in calcineurin-dependent gene expression will need to be investigated. Indeed, in skeletal muscle, calcineurin can regulate the expression of some mitochondrial genes [44]. Whether such a mechanism exists in β-cells remains, to our knowledge, unknown. We hypothesise that the expression of unidentified mitochondrial proteins are altered in RCAN1^ox^ β-cells, resulting in reduced nucleotide transport and hyperpolarisation of the mitochondrial membrane, to reduce glucose-stimulated insulin secretion.

Our patch clamp studies provide a functional link between RCAN1-induced mitochondrial dysfunction and reduced insulin secretion. The fact that tolbutamide had the same effect in WT and RCAN1^ox^ β-cells, but that high glucose had a reduced effect on membrane depolarisation in RCAN1^ox^ cells is clear evidence that glucose-induced K_ATP_ channel closure is hindered in RCAN1^ox^ β-cells. Biphasic glucose stimulated insulin secretion is reduced in T2D islets [25,45] and we demonstrate here the same phenotype in RCAN1^ox^ islets. Unaltered insulin sensitivity and glucagon release in RCAN1^ox^ mice provides further support that the reduced *in vivo* GSIS we observe in RCAN1^ox^ mice is due to β-cell failure. Glucose metabolism raises the cellular ATP/ADP ratio in β-cells to close K_ATP_ channels, depolarise the plasma membrane and open voltage-gated Ca^2+^ channels to trigger insulin granule exocytosis. Whether membrane depolarisation is reduced in T2D β-cells as we observe in RCAN1^ox^ β-cells is unknown, but might be assumed given the reduced glucose oxidation [25] and ATP production [4] reported in human T2D islets.

The use of whole cell capacitance to provide 3mM ATP to these β-cells demonstrates that a lack of ATP drives the low exocytosis phenotype in RCAN1^ox^ β-cells. The fact that we were able to rescue this exocytosis phenotype with a single molecule (i.e.; ATP) is a very strong indicator that low ATP levels are responsible for the secretory defect observed in RCAN1^ox^ β-cells. The earliest phase of exocytosis represents release of docked insulin granules, while the slower second phase requires granule recruitment from the reserve pool in an ATP-dependent manner [24]. Our capacitance data shows the readily releasable vesicle pool is not affected in RCAN1^ox^ β-cells, which is unsurprising given insulin granule localisation is unchanged in RCAN1^ox^ β-cells [15]. It appears that RCAN1^ox^ β-cells produce enough ATP under basal conditions for vesicle docking and priming to occur, and defects in exocytosis are only observed under stimulated conditions where rapid ATP generation is required. Our data also demonstrates that perforated patch clamp should be used in such experiments so as to avoid artefacts produced by washout of cytoplasmic second messengers.

This genetic screening approach we have developed combining different trisomy 21 mouse models with whole genome data from human T2D islets has identified a potential regulator of β-cell mitochondrial dysfunction in T2D. This work goes well beyond previous findings related to the function of RCAN1 by identifying it, through an unbiased multi-centre screening approach, as a lead candidate in the control of whole body glucose metabolism and in the β-cell dysfunction which is central in humans to the transition from insulin resistance to T2D. Furthermore, our data demonstrating that *RCAN1* methylation is reduced at multiple sites in human T2D β-cells and that methylation status at these sites correlates with *RCAN1* expression is further validation of our screening approach and provides a mechanistic pathway that clearly explains how RCAN1 expression changes in T2D β-cells. Similarly, such an approach could be applied to other human health disorders with phenotypes shared with DS individuals. Given the large number of gene changes observed in complex human diseases like T2D, such an approach could aid in deciphering the complex data sets now being obtained with ever improving genetic search tools. An example of this provided by this present study is insulin resistance, as the hypoglycaemic status of Tc1 mice indicates that chromosome 21 genes unique to this DS mouse model may improve some aspects of insulin sensitivity. This may have clinical applications for T2D research also, given the importance of insulin resistance in the pathogenesis of this disease.

## Materials and Methods

### Mouse lines

3–5 month old male mice were used in this study with approval from the individual institutional animal welfare committees at each site. Dp(16Cbr1-ORF9)1Rhr (called Ts1Rhr) [46], Dp(16)1Yey (called Dp16) [47] and Ts65Dn [48] mice were maintained on a B6EiC3Sn/J background. Tc1 mice (called Tc1TybEmcf) [49] were bred by crossing female Tc1 mice to male (C57BL/6JNimr x 129S8/Nimr) F1 mice. Mice homozygous for the diabetes spontaneous mutation (Lepr^db^), named here as db/db mice, were maintained on a C57BL/KsJ background. RCAN1^ox^ mice are transgenic C57BL/6xCBA mice stably overexpressing human RCAN1.1 [20]. While multiple RCAN1^ox^ founder lines were originally generated, only a single line was used for this study. We observed no overt differences between the three RCAN1^ox^ lines initially created, but these investigations were unrelated to glucose metabolism.

### Ethics statement

Use of human islets was approved by the ethics committee at Lund University and University of Adelaide. In Adelaide, human islets were obtained from heart beating organ donors through the Australian Islet Consortium (RAH 100205b and St Vincent’s Hospital Melbourne (SVH HREC-D 103/05). Consent for donated human pancreata for research in all cases was obtained through Donate Life Australia. In Sweden, informed consent for organ donation for medical research was obtained from pancreatic donors or their relatives in accordance with the approval by the regional ethics committees (173/2007) in Lund and Uppsala, Sweden. Use of mice was approved by the Flinders University, University of California Irvine, Garvan Institute, MRC Harwell and University of California San Diego Animal Ethics Committees. Mice were euthanized via overdose inhalation of isoflurane. All animal research at Flinders University was approved by the Flinders University Animal Welfare Committee (797–11, 620/07) following guidelines issued by the National Health and Medical Research Council of Australia. Approval at USCD was provided by the UCSD Institutional Animal Care and Use Committee (S09315) and followed guidelines provided by that body. All animal work at MRC was carried out with the approval of the Ethical Review Board and under Licence from, and following guidelines provided by, the UK Home Office. At the Garvan Institute, procedures were approved by the Garvan Institute/St.Vincent’s Hospital Animal Experimentation Ethics Committee (14_21), following guidelines issued by the National Health and Medical Research Council of Australia. At UCI, animal experiments involving mice were approved by the University of California Irvine (UCI) institutional animal care and use committee (IACUC) (approval number: 2008–2779) following guideline provided by that body.

### Fasting glucose and glucose tolerance measurements

Blood was collected from mice fasted for 6 hours. For Ts65Dn, Dp16 and Ts1Rhr mice, blood was obtained from tail tips and glucose measured using a One-Touch Ultra Blood Glucose Monitoring System (LifeScan, Milpitas, CA, USA) or Contour Blood Glucose Monitoring System (Bayer HealthCare, Mishawaka, IN, USA). For Tc1 mice, blood was collected from the retro-orbital sinus and measured using an AU680 clinical chemistry analyser (Beckman Coulter, Brea CA, USA). After 12–16 hour overnight fasting, glucose tolerance was measured by administering intra-peritoneal glucose (2.0 g/kg).

### RNA sequencing

Islets from 89 cadaver donors (77 control and 12 T2D) of European ancestry were provided by the Nordic Islet Transplantation Programme and processed as described [8].

### Human islets

Pancreases were removed from heart beating deceased donors and disaggregated by infusing the ducts with cold collagenase (NB1 GMP grade from SERVA, Heidelberg, Germany). Dissociated islet and acinar tissue were separated on a continuous Biocoll (Biochrom AG, Berlin) density gradient (polysucrose 400 and amidotrizoic acid) on a refrigerated apheresis system (Model 2991, COBE Laboratories, Lakewood, CO.)

### Mouse pancreatic islets and MIN6 β-cell line

Pancreatic islets isolated from age matched, male C57BL/6 × CBA wild type (WT) and transgenic mice stably overexpressing human RCAN1.1 (RCAN1^ox^) [20] were used in all experiments. Experiments were approved by the Flinders University Animal Welfare and Institutional Biosafety Committees. Mice were killed by an anaesthetic overdose of isoflurane and islets isolated as previously described [15]. MIN6 cells, an insulinoma cell line derived from a transgenic mouse expressing the large T-antigen of SV40 in pancreatic β-cells (passage number 37–39), were grown in Dulbecco’s Modified Eagles Medium (DMEM) media (11.1 mM glucose, 15% fetal calf serum) at 37°C in 5% CO_2_.

### Quantitative real-time PCR

Islet RNA was extracted using QIAshredder columns and an RNAeasy minikit (Qiagen). All RNA samples were subjected to a DNAse treatment to remove any genomic DNA (TURBO DNA-free kit, Life Technologies) prior to reverse transcription (Omniscript, Qiagen). Quantitative real-time PCR analysis was carried out in triplicate utilising SyBR-Green (Qiagen). All results were normalised to β-actin expression which was used as a house-keeping gene. Mean normalised expression values and fold gene expression were calculated using Qgene Module software.

### SeahorseXF analysis

The respiratory responses of islets isolated from wild type and RCAN1^ox^ mice were assessed using the Seahorse XF24 Flux Analyser (Seahorse Bioscience). Islets were washed in DMEM (3mM glucose, 1mM pyruvate, 1mM glutamate, 1% fetal bovine serum) and approximately 50 islets were added to each well of a 24-well XF24 islet capture microplate. Islets were incubated at 37°C in a non-CO_2_ incubator for 1hr prior to bioenergetics assessment. Six basal oxygen consumption rate (OCR) measurements were performed using the Seahorse analyser and measurements were repeated following injection of 20mM glucose, 5μM oligomycin, and 1μM Antimycin A. Respiratory parameters of mitochondrial function were calculated as described previously [50]. Non-mitochondrial respiration was subtracted from all mitochondrial respiration parameters. Following the assay, protein content of each well was assessed by standard BCA assay (Thermo Fisher). Mitochondrial respiration was normalised to total islet protein content.

### Western blot analysis

25 μg of islet or mitochondrial protein lysates were separated on a Criterion-TGX stain-free gel (Bio-Rad). Immunoreactive proteins were visualised on a Fuji-Film LAS4000 and Gel-Doc Ez-Imager (Bio-Rad). Primary antibodies; anti-RCAN1 (1:200, Sigma Aldrich), anti-TOM20 (1:1000, Jackson Laboratories), anti-β-actin (1:200, Sigma Aldrich), anti-NDUFA9 (1:1000, Sigma Aldrich), anti-SDHA (1:2000, Sigma Aldrich), anti-CORE1 (1:1200, Sigma Aldrich) and anti-Opa1 (1:1500, Sigma Aldrich). Secondary antibodies; donkey anti-rabbit horse-radish peroxidase (HRP) (1:100, Life Technologies) and donkey anti-mouse HRP (1:100, Life Technologies).

### Electrophysiology

Whole-cell patch clamp recording was performed using an EPC‐10 patch clamp amplifier and PatchMaster software (HEKA Electronik GmbH). Patch pipettes were pulled from borosilicate glass and fire polished, with resistance of 3–5 MΩ. Patch clamping was performed in the perforated patch configuration for capacitance measurements, with internal solution containing (mM): 140 CsCl, 2 MgCl_2_, 5 EGTA, 0.5 CaCl_2_ and 10 Hepes, adjusted to pH 7.2, and with 240 μg ml^−1^ amphotericin B. For whole cell capacitance measurements, amphotericin was removed and 3mM ATP added. External solution contained (mM): 140 NaCl, 5 Hepes, 2 MgCl_2_ and 10 CaCl_2_ and, adjusted to pH 7.4 with NaOH. Capacitance measurements utilized the Lock-in module of the PatchMaster software, with capacitance change measured in response to 10 voltage steps to 10mV of 500 ms duration at 1Hz from a resting membrane potential of −80 mV. Internal solution for measurement of K^+^ currents and membrane potential was (mM): 10 NaCl, 145 KCl, 10 Hepes, 1 MgCl_2_, 1 EGTA, adjusted to pH 7.2. External solution was (mM): 140 NaCl, 2.8 KCl, 10 Hepes, 1 MgCl_2_, 2 CaCl_2_ adjusted to pH 7.4. Cells were identified on their response to high glucose and/or having a whole cell capacitance over 5pF. Experiments were carried out at 22–24°C.

### ATP content

ATP content of groups of 80 islets in the presence of 20 mM glucose was measured using a commercially available bioluminescent ATP quantitation kit (Sigma Aldrich) as per the manufactures instructions. ATP content was normalised to total protein content.

### Insulin and glucagon measurements

Insulin secretion assays from isolated islets were carried as previously described [15]. For some experiments, potassium carboxyatractyloside (200 μM) or methyl-succinate (10 mM) were added. Plasma glucagon was measured from mice fasted for 2 hours using an ELISA (Crystal Chem, USA). The *in vivo* GSIS measurements were undertaken on mice fasted for 2 hours and 2mg/g body weight of glucose (PharmaLab, Australia) was injected (i.p.) into mice and blood glucose measured at various interval from 0 to 60 minutes via a tail bleed. Insulin was quantified using an Ultra Sensitive Mouse Insulin ELISA Kit (Crystal Chem, USA) as per the manufacturer’s instructions for a low range assay. Absorbance was measured at 450 nm and 620 nm using a BIOMECK-3000 micro-plate reader (Beckman-Coulter, USA) and MultiMode detection software.

### Insulin tolerance test

Insulin tolerance tests were carried on mice fasted for 1 hour and 0.75 units/kg body weight insulin (Novo Nordisk, Australia) was injected (i.p.) into 40 day old mice. Blood glucose was measured via a tail bleed using an ACCU-CHEK Performa glucometer (Roche Diagnostics, Australia).

### Mitochondrial analysis

Mitochondrial volume was calculated from electron micrographs obtained as previously described [15]. Total mitochondrial volume per cell was calculated from these images using ImageJ image analysis software (National Institute of Health, Bethesda, MD, USA) after subtraction of the nuclear volume. This was calculated as both absolute volume and the volume fraction of the entire β-cell. Relative volume was also calculated from isolated cells loaded for 30 min with tetramethylrhodamine methyl ester (TMRM, 1μM). Images were analysed using the masking function in the Image J digital image analysis software (National Institute of Health, Bethesda, MD, USA). Mitochondrial membrane potential was measured as the mean cell fluorescence of cells loaded with TMRM. Images were captured under identical conditions for all groups and data presented as mean TMRM fluorescence in each group.

### RCAN1 methylation analysis

DNA methylation profiling of human pancreatic islets was performed at the SCIBLU genomics center at Lund University with the Infinium HumanMethylation450 BeadChip (Illumina, Inc., San Diego, CA). The experimental and bioinformatics analyses have previously been described [18]. In short, DNA from human pancreatic islets was bisulfite converted using the EZ DNA Methylation Kit D5001 (Zymo Research, Orange, CA) according to the manufacturer’s instructions. Bisulfite converted DNA was amplified, fragmented and hybridized to the BeadChips following the standard Infinium protocol. T2D islet samples were randomized across the chips and all samples were analyzed on the same machine by the same technician to reduce batch effects. The DNA methylation data were exported from GenomeStudio and Bioconductor and the lumi package were used for further analyses. Individual probes were filtered based on their mean detection *P*-value and those with a *P*-value > 0.01 were excluded from further analysis. M-values were calculated from β-values using the following equation: M = log_2_ β-value / (1-β-value) and were then used for further statistical analysis. To identify differences in DNA methylation between T2D and non-diabetic islets a linear regression model was used including batch, gender, BMI, age, islet purity and days of culture as covariates and DNA methylation as the quantitative variable. As the β-value is easier to interpret biologically, M-values were converted to β-values when describing the results and creating the figures.

### Mitochondrial RCAN1 localisation

Cells were incubated with MitoTracker Red (1μM) at 37°C for 15 min and then fixed with 4% formaldehyde for 15 min at 22°C. The cells were stained with anti-RCAN1 antibody (1:100) and FITC conjugated antibody (1:100) as previously described [51]. The cells were viewed under laser scanning confocal microscope (Olympus, FV1000-IX81 Japan) using a 40X oil immersion lens. Multitrack scanning mode was used to record single- and double-labelled cells.

### Statistical analysis

Parametrically distributed data were analysed using a Student’s unpaired *t* test and a Mann-Whitney *U* test was used to analyse nonparametric data sets. Statistical significance was *p <* 0.05. All data are shown as mean ± SEM.

## Supporting Information

S1 Fig (A) Correlation between BMI of donors and RCAN1 expression in isolated human islets. (B) RCAN1 expression in islets isolated from individuals with a Hba1c level greater than 6.5% (black symbols) compared to islets from individuals with an Hba1c range of 6–6.5 (red symbols) and with a Hba1c level less than 6 (blue symbols). Correlation between RCAN1 expression and (C) triglycerides and (D) body weight in different diabetic mouse models.
(TIF)

S2 Fig Depiction of the genomic structure of RCAN1.1 and RCAN1.4. Exons are shown as boxes and their respective numbers provided. Lines separating boxes represent introns. Length of exons and introns are not to scale. Multiple Nuclear factor of activated T-cells (NFAT) binding sites exist upstream of exon 4.
(TIF)

S3 Fig (A) OCR due to H^+^ leak and (B) basal mitochondrial OCR are significantly lower in RCAN1^ox^ (n = 5 experiments) compared to wild type islets (n = 6 experiments). (C) OCR due to ATP turnover is not statistically different between the two groups (p = 0.08).
(TIF)

S4 Fig The current voltage relationship in (A) WT (n = 6) and (B) RCAN1^ox^ (n = 6) β-cells demonstrates reduced K^+^ current in the presence of high glucose. Inset: zoomed view of approximate reversal potential in these recordings shows a shift in WT but not RCAN1^ox^ cells. Similar data with tolbutamide in (C) WT (n = 7) and (D) RCAN1^ox^ (n = 5) β-cells shows similar K^+^ current reduction and shift in reversal potential.
(TIF)
