# Supplementary figures and images for: A Syntenic Cross Species Aneuploidy Genetic Screen Links RCAN1 Expression to β-Cell Mitochondrial Dysfunction in Type 2 Diabetes
Source: PLoS Genet. 2016 May 19;12(5):e1006033. doi: 10.1371/journal.pgen.1006033 (PMC4873152; doi:10.1371/journal.pgen.1006033)

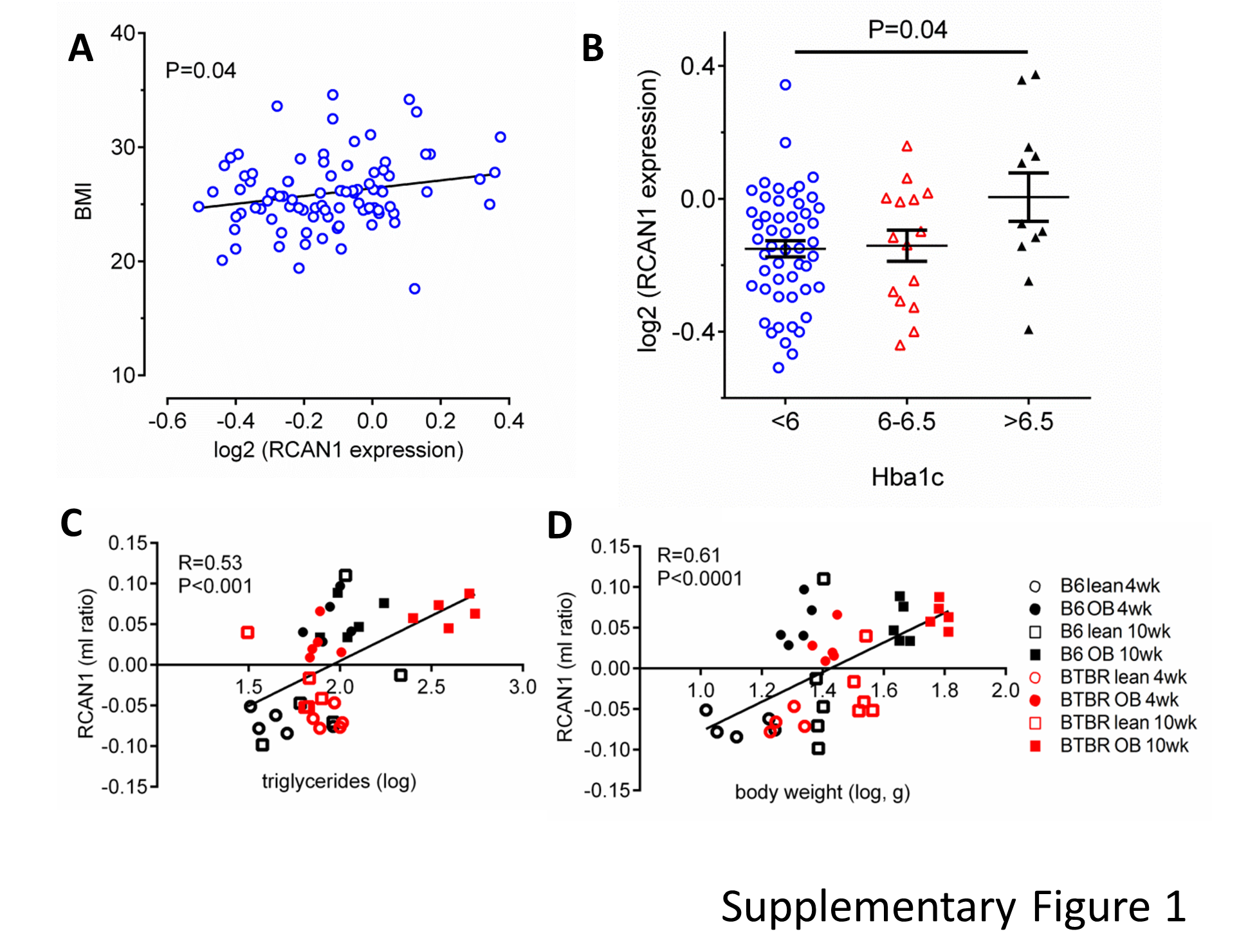

Supplement: S1 Fig — (A) Correlation between BMI of donors and RCAN1 expression in isolated human islets. (B) RCAN1 expression in islets isolated from individuals with a Hba1c level greater than 6.5% (black symbols) compared to islets from individuals with an Hba1c range of 6–6.5 (red symbols) and with a Hba1c level less than 6 (blue symbols). Correlation between RCAN1 expression and (C) triglycerides and (D) body weight in different diabetic mouse models. (TIF) [file pgen.1006033.s001.TIF]

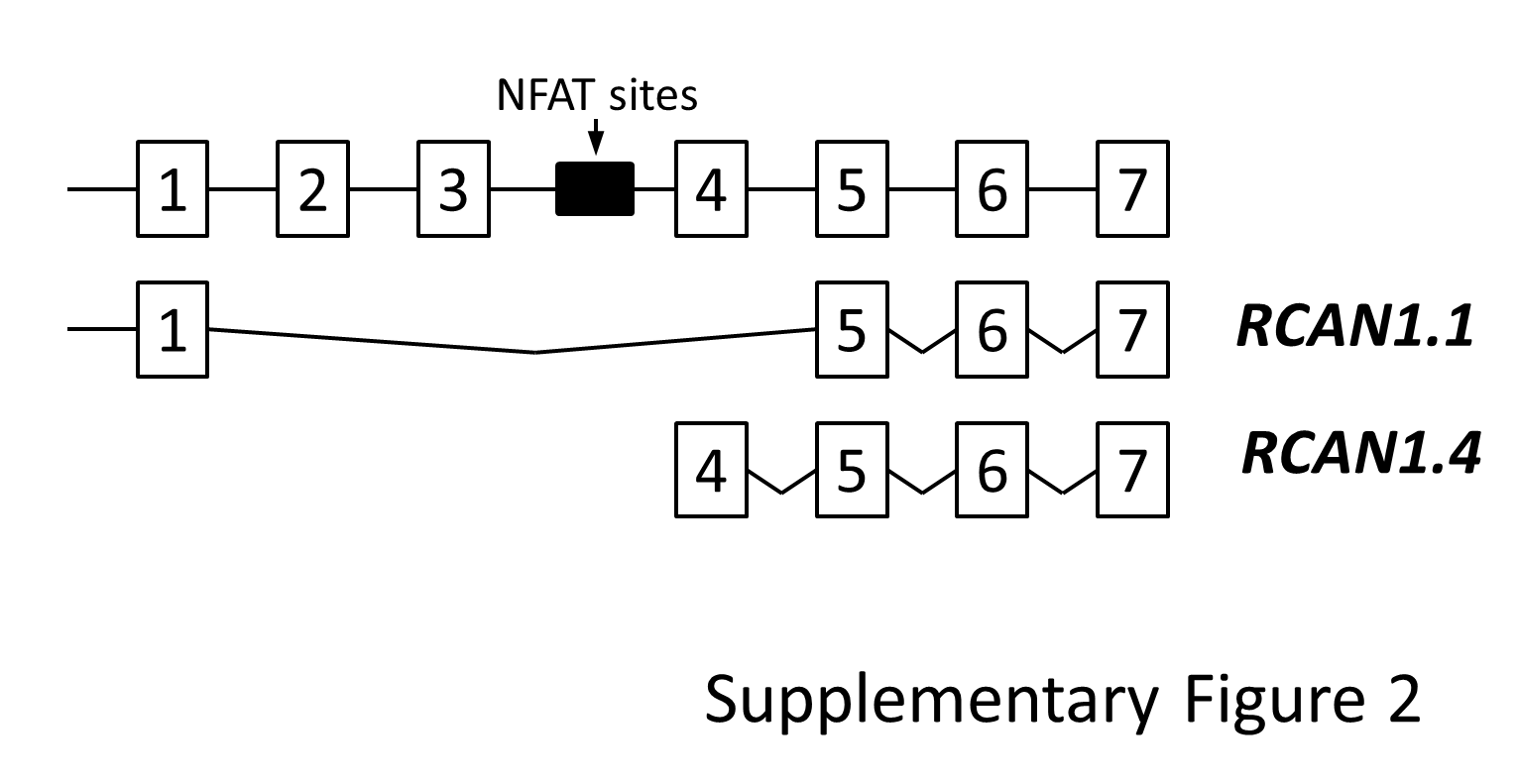

Supplement: S2 Fig — Exons are shown as boxes and their respective numbers provided. Lines separating boxes represent introns. Length of exons and introns are not to scale. Multiple Nuclear factor of activated T-cells (NFAT) binding sites exist upstream of exon 4. (TIF) [file pgen.1006033.s002.tif]

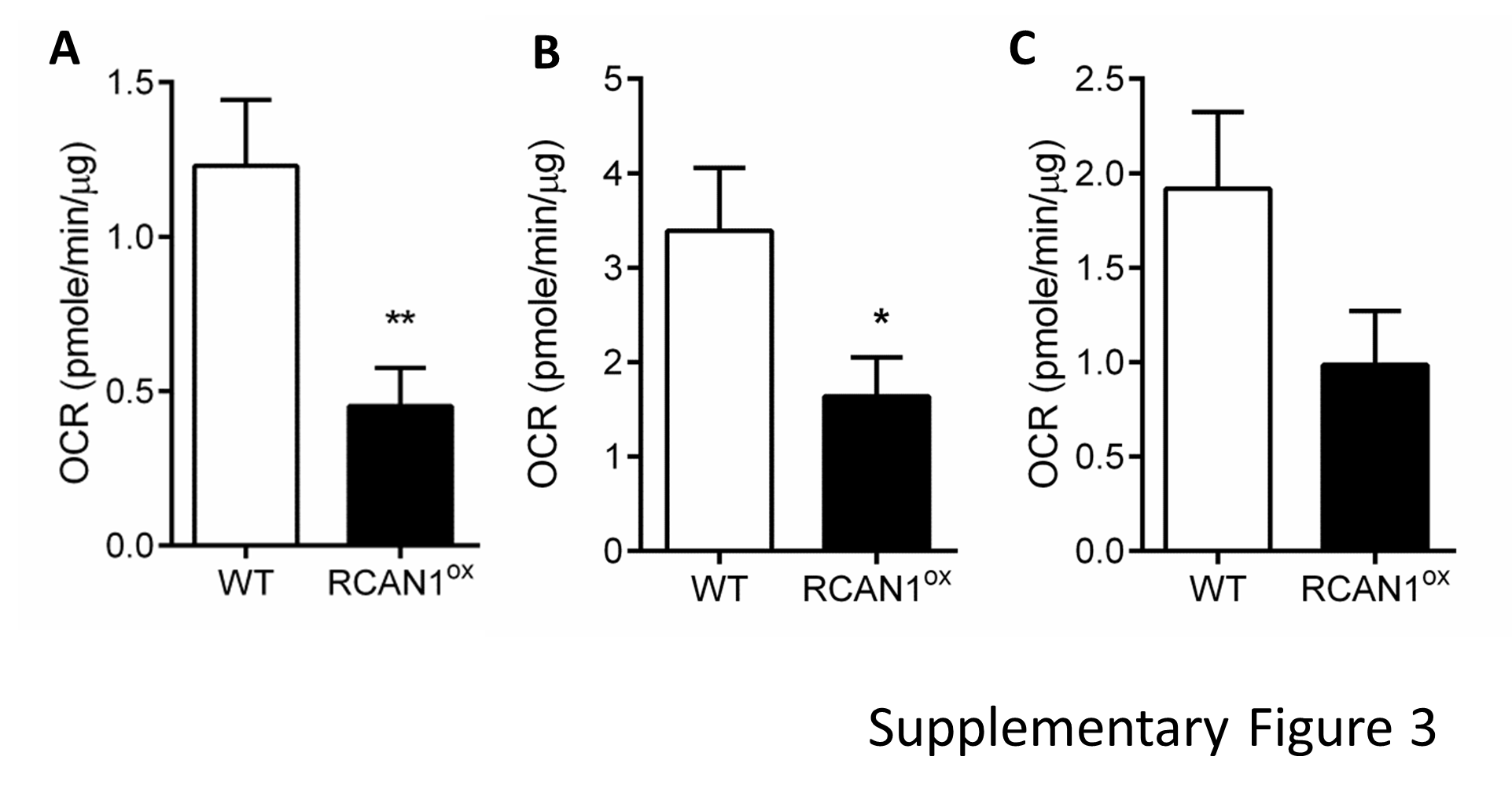

Supplement: S3 Fig — (A) OCR due to H+ leak and (B) basal mitochondrial OCR are significantly lower in RCAN1ox (n = 5 experiments) compared to wild type islets (n = 6 experiments). (C) OCR due to ATP turnover is not statistically different between the two groups (p = 0.08). (TIF) [file pgen.1006033.s003.TIF]

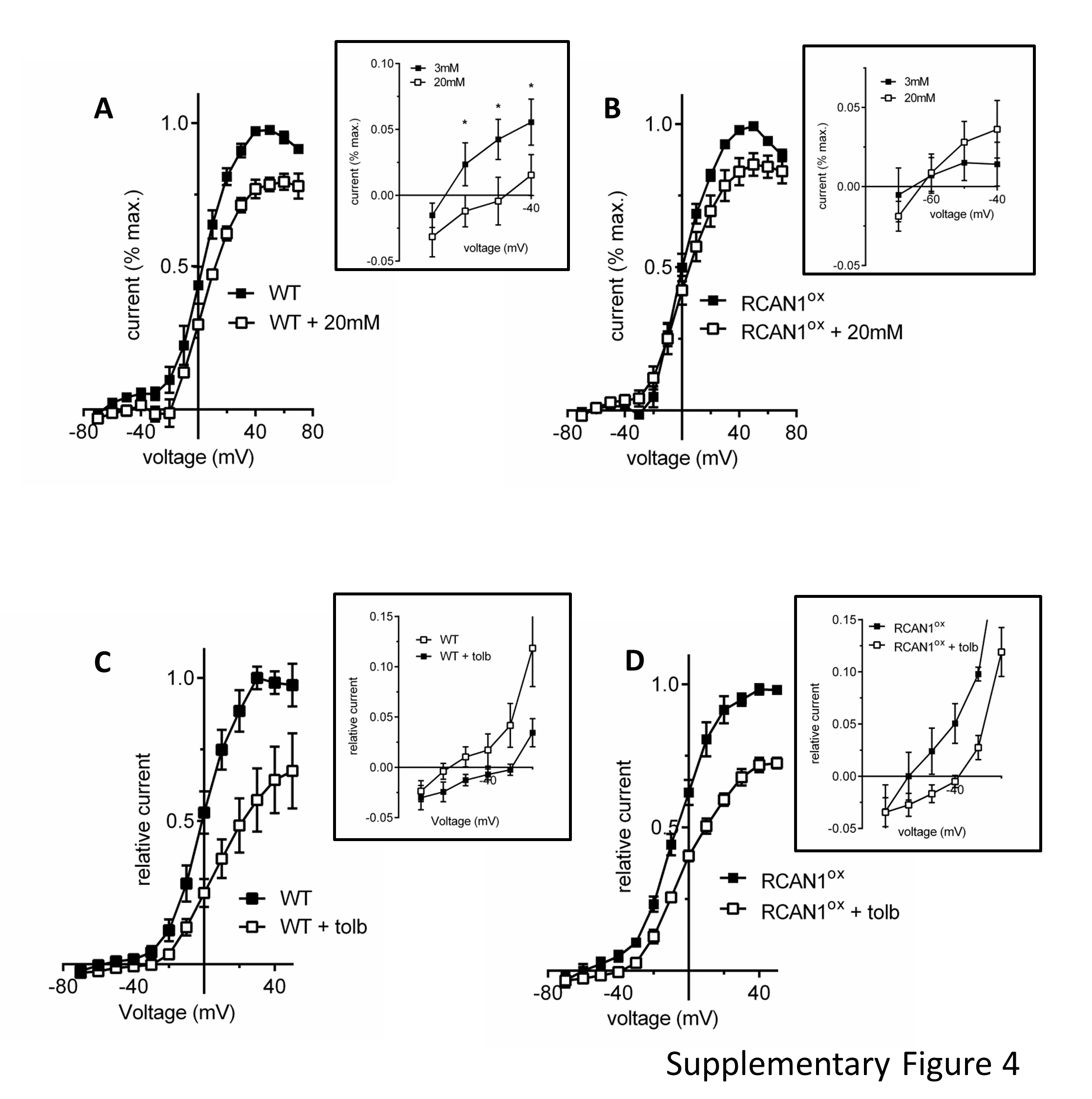

Supplement: S4 Fig — Inset: zoomed view of approximate reversal potential in these recordings shows a shift in WT but not RCAN1ox cells. Similar data with tolbutamide in (C) WT (n = 7) and (D) RCAN1ox (n = 5) β-cells shows similar K+ current reduction and shift in reversal potential. (TIF) [file pgen.1006033.s004.TIF]
